# An Interactive Wireless Communication System for Visually Impaired People Using City Bus Transport

**DOI:** 10.3390/ijerph110504560

**Published:** 2014-04-25

**Authors:** Hsiao-Lan Wang, Ya-Ping Chen, Chi-Lun Rau, Chung-Huang Yu

**Affiliations:** 1 Department of Physical Therapy and Assistive Technology, National Yang-Ming University, No.155, Sec.2, Linong Street, Beitou District, Taipei 11221, Taiwan; E-Mails: hsiaolan88@gmail.com (H.-L.W.); chyu@ym.edu.tw (C.-H.Y.); 2 Department of Physical Medicine and Rehabilitation, Shuang Ho Hospital, Taipei Medical University, No.291, Zhongzheng Road, Zhonghe District, New Taipei City 23561, Taiwan; E-Mail: yapingchen21@gmail.com

**Keywords:** wireless communication, visual impairment, public transportation

## Abstract

Visually impaired people have difficulty accessing information about public transportation systems. Several systems have been developed for assisting visually impaired and blind people to use the city bus. Most systems provide only one-way communication and require high-cost and complex equipment. The purpose of this study is to reduce the difficulties faced by visually impaired people when taking city buses, using an interactive wireless communication system. The system comprised a user module and a bus module to establish a direct one-to-one connection. When the user inputs 4-digit numbers, the user module immediately sends out the information. If the bus module receives the matched bus number, it buzzes and the warning LED flashes to notify the bus driver that someone is waiting to board on the bus. User tests were conducted by two visually impaired people in a simulated vehicle and a city bus. The success rate of interactive wireless communication, recognizing the arrival of the bus and boarding the correct bus reached 100% in all of the tests. The interactive wireless communication aid system is a valid and low-cost device for assisting visually impaired people to use city buses.

## 1. Introduction

The use of public transport is vital to the productivity and independence of visually impaired people. Helping visually impaired people use public transport can increase their chances of education and employment and reduce the financial burden on their families [1,2]. In most physical environments, the visually impaired have difficulty accessing information about transport stops, terminals, vehicles, schedules, maps, and directories, which prevent them from using public transport effectively. According to a survey in Taiwan on the living demands of disabled people, using public transport was the most critical problem for the visually impaired, amounting to 71.04% of 602 visually impaired people [3]. The survey results showed that only 14% of visually impaired people used public transport (city bus, mass rapid transit, train, *etc.*).

Knowing the location of the bus stop and the time when the bus arrives are two common difficulties faced by the visually impaired. Some position navigation systems have been developed to solve the problem of locating bus stops [4,5,6]. Advanced public transportation services (APTS), including bus dynamic information display systems with the Global Positioning System (GPS) technology, have been developed by many countries. APTS can be equipped with the bus-stop voice reporting systems to provide more information to visually impaired people on the arrival of the bus they want to board. Some APTS combining special handheld devices were designed to provide the electronic orientation and dynamic information for visually impaired people. Examples include Apex in Czech [7], Bus-ID in German [8], iBus in Canada [9], NOPPA in Finland [10], PAVIP (Personal Assistant for Visual Impaired People) in Switzerland [11], and Quo Vadis in Austria [12]. Recently, smart phones become more and more widespread. Markiewicz and Skomorowski proposed an aid system using mobile phones (Java and GPS-function enabled) as the user modules [13]. Some smart phone apps providing local bus information through special or voice interface are developed for the visual impaired like Geogiephone [14], PT Guide [15], and Voice@Bus [16].

In Taiwan, the Taipei city government once utilized an experimental system named “broadcasting bus”, announcing the bus number upon arriving at the bus terminal, to serve the visually impaired in 2002 [17]. Now there are about 300 bus routes, 4,000 buses, and 5,000 bus stops in Taipei city [18,19]. The APTS named “e-bus system” started to be deployed in Taipei city since 2005. The total budget was about 7 million U.S. dollars [20]. The GPS position of the buses was transmitted to a server at a control center by general packet radio service (GPRS) technology, and the related information was transmitted to the intelligent bus terminals by GPRS technology immediately. The number and the waiting time of the buses were shown on the light-emitting diode (LED) screen at the smart bus stops. Totally 1000 smart bus stops, 20 percent of all bus stops, is planned to build. A pilot survey of 400 passengers in 1998 showed that the smart bus stops are not adequate when equipped only with the voice reporting system because voice information was confusing when many buses approached the same terminal at the same time [21].

The APTS with bus-stop voice reporting system and/or handheld devices might not provide a comprehensive solution to this important issue. A survey on visually impaired people showed that it would be very helpful if somebody could alert bus drivers about their boarding on buses [22]. Various systems have been developed for visually impaired and blind people to communicate with bus drivers. Mehra *et al.* developed a user-triggered bus identification system in 2010 [23]. The user could select a particular bus and send signals by user module, and then a small bulb starts flickering in the driver’s control panel. This system offered only one-way communication. Bischof *et al.* developed a wireless local area network (WLAN) communication system named NAVCOM [24]. The authors proposed that blind people need a feedback to acknowledge whether the bus drivers get the original message. Another bus identification system designed by El Alamy *et al.* involves a bus station controller to recognize users and send signals to buses with radio frequency [25]. This system will announce the information of the bus number when there is a 2-meter distance between the bus and the bus station. In Taipei, bus drivers are inclined to leave the bus stop as soon as possible because most time several buses simultaneously arrive at the same bus stop. Visually impaired people are often ignored at bus stops if no one informs bus drivers about waiting passengers behind. The purpose of the present study is to reduce the difficulties faced by visually impaired people when taking buses with interactive wireless communication design. The interactive feedback mechanism would allow visually impaired people and bus drivers to receive the transmitted signals from each other and improve the success rate of boarding correct buses.

## 2. Materials and Methods

### 2.1. Participants

The participants in this study were two visually impaired people, participant A was one 52 year-old male and participant B was one 50 year-old female. The male subject had been totally blind for 20 years as a result of retinitis pigmentosa, without any other disability. The female subject’s total blindness was congenital without any other disability. Both participants were independently mobile with the use of a guide cane.

### 2.2. System Development

In a real environment, the interaction between visually impaired people and bus drivers is a many-to-many relationship rather than a one-to-one. For technological simplicity, this system used a one-to-one interactive communication system. The collision of communication data over the one-to-one interactive wireless transmission leads to the poor stability and low accuracy of wireless communication. Wireless communication technology that do not experience collision include time division multiple access (TDMA), code division multiple access (CDMA), and frequency-division multiple access (FDMA). This study primarily refers to the principles and concepts of FDMA, that is, the signal communicating through two distinct frequency bands: 434 MHz and 315 MHz.

**Figure 1:**
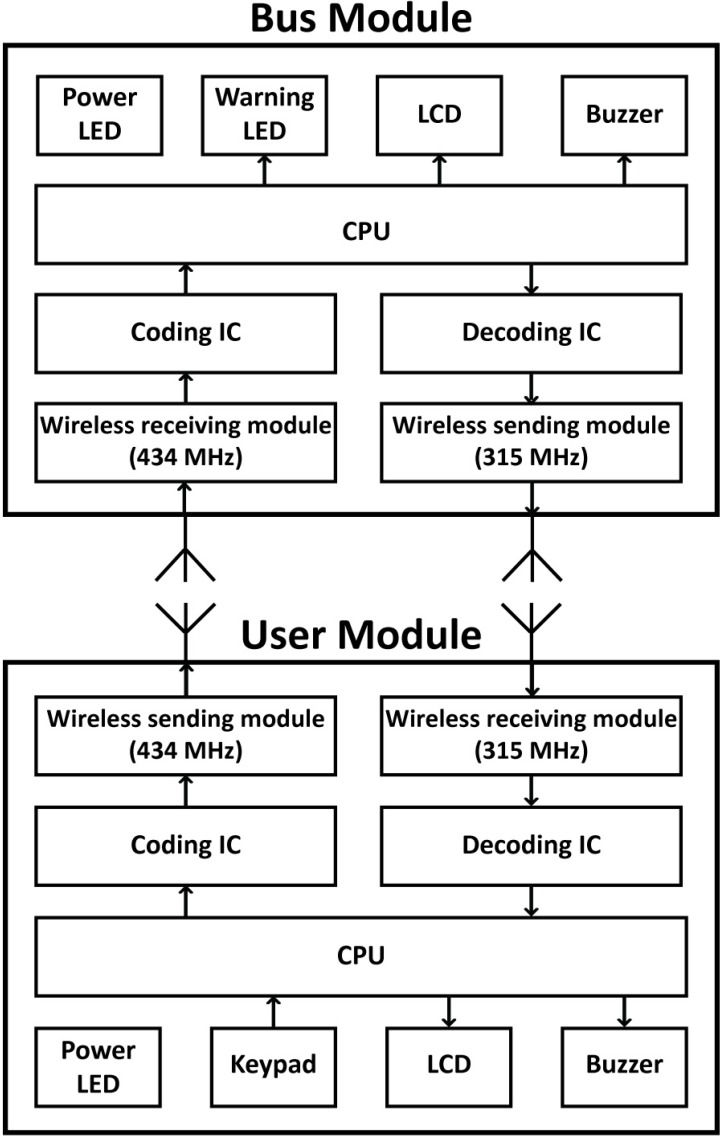
System framework of the user module and the bus module.

**Figure 2:**
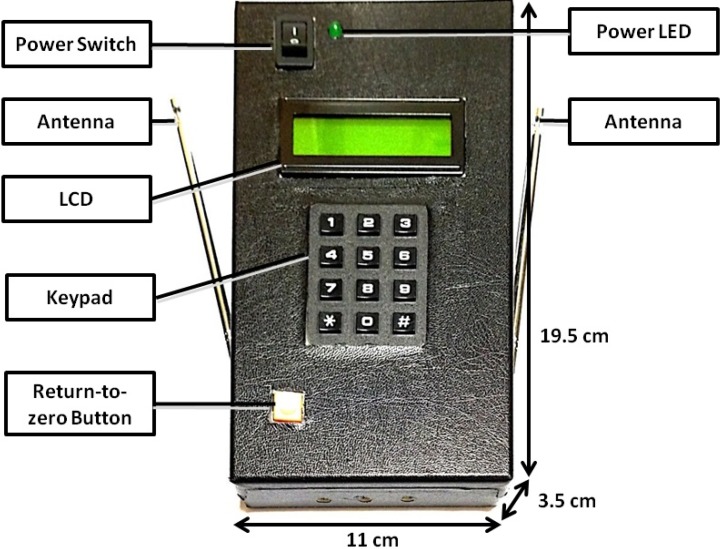
User module.

**Figure 3:**
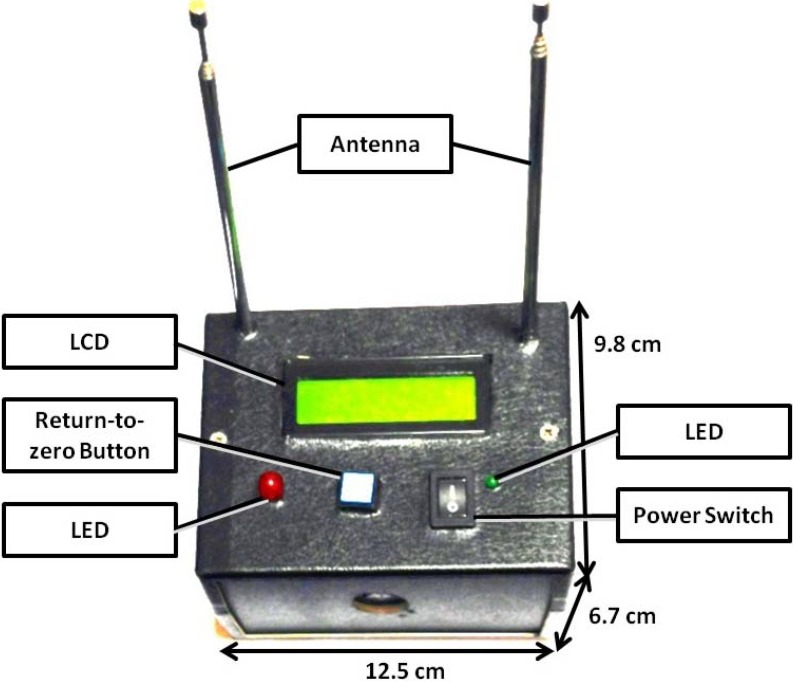
Bus module.

The interactive wireless communication system would have two modules: a user module for the visually impaired and a bus module for bus drivers (Figure 1). The hardware was developed according to the interactive design. The user module (Figure 2) consists of a single-chip CPU (PSoC^®^CY8C27443), a wireless transmission module (434 MHz), a wireless receiving module (315 MHz), a coding IC (HT-12E), a decoding IC (HT-12D), a buzzer, a keypad, an LCD, and a power LED. The bus module (Figure 3) consists of a single-chip CPU (PSoC^®^CY8C27443), a wireless transmission module (315 MHz), a wireless receiving module (434 MHz), a coding IC (HT-12E), a decoding IC (HT-12D), a buzzer, an LCD, a warning LED, and a power LED. When the user module sends out a signal, it is transmitted through the 434 MHz band to the bus module, which then replies through the 315 MHz band to the user module. In this way, the problem of wireless data collisions between users is avoided.

A prototype of user module was tested by both participants. After testing the prototype, the participants suggested several areas of improvement, including the device’s size, operational inconvenience—particularly the power switch and button settings, and low buzz volume. The devices were improved according to the users’ feedback. The total price of our system is about 120 U.S. dollars.

### 2.3. Module Interface and Interactive Wireless Communication Design

When the user module power is switched on, the power LED is on, and the LCD displays “Bus No?” to prompt the user to input the preset bus numbers. The module can store 16 sets of 4-digit numbers, for example: #074, *074, and *001. The sign # and * represent the traveling direction of city buses, and the number behind the sign represents the bus number. The indication of the direction is necessary because buses with the same number traveling in different directions will receive the signal at the same time. When the user has successfully input one of the preset 4-digit numbers, the module immediately sends out the information through the wireless transmission module continuously. Because this system was designed to assist visually impaired people, the keypad has audible tones. Additionally, if the input number was not the same as the preset number, the buzzer of the user module emits a specific sound to inform the user that the number is incorrect. However, if the input number is correct, no sound is emitted. When the target bus is coming, and the signal of the correct bus number is received, the buzzer is activated and the bus number is displayed on the LCD to inform the user that the bus is arriving.

16 sets of 4-digit numbers representing bus traveling directions and bus numbers are also preset in each bus module. When the power of the bus module is switched on, the power LED is on, and the LCD displays “Bus Number: #xxx” or “Bus Number: *xxx”. The sign # and * represent the traveling direction of city buses, and the xxx behind the sign represents the bus number. If the bus module receives the correct bus number, the bus module buzzes and the warning LED flashes, and the LCD shows “Attention!” to notify the bus driver that someone is waiting to board that particular bus. The bus module also sends the same bus number back to the user module at the same time. When the bus drivers have taken on the passenger, they can press the return-to-zero button to turn off the warning LED and the buzzer. Then the bus module is available for the next passenger. The user can press the return-to-zero button to turn off the warning LED and the buzzer after the arrival of the bus. Then the user module is available for inputting the next set of numbers.

### 2.4. Outcome Measure

A basic requirement of this system was the wireless communication distance. If the wireless communication distances were shorter than buses’ stopping distances, bus drivers would fail to stop buses for visually impaired people. The stopping distance of a car is equal to the reaction time plus the braking distance. Stopping distance can be affected by vehicle weight, weather, road condition, and speed. The speed limit for city buses in Taipei is 40 km/h, but most buses always travel at a speed less than 40 km/h and slow down when approaching bus stops. Because the users were waiting at bus stops, we decided to keep the simulated vehicle in tests at 30 km/h. The stopping distance of a bus at 30 km/h on dry roads is approximately 12.1 meters [26], and this was the parameter for our tests. Conversely, if the wireless communication distance was too long, it was possible that the bus driver would have received the information from a waiting user too early. To avoid such situations, the maximum transmission distance of this system must be less than 100 meters.

The following tests measured the wireless communication distance, the success rate of the interactive wireless communication, and visually impaired people recognizing arrival of the bus and boarding the correct bus.

### 2.5. Test Procedure

Test 1 was conducted on a city road with few vehicles, and measured the accuracy of the interactive wireless communication under various weather conditions. One experimenter held the user module and input the bus numbers, while the other experimenter took the bus module and drove a motorcycle at 30 km/h to the former from a long distance away. When the bus module buzzed and the warning LED flashed, the distance was recorded. This test was repeated five times in sunny, cloudy, and rainy weather.

Test 2 was conducted on a campus road, with few vehicles present, and in sunny weather. Before the tests, it took the two participants approximately three to five minutes to learn how to use the user module. The participants held the user module at a fixed location. The experimenter drove a sports utility vehicle (SUV), equipped with the bus module, as a simulated bus. The starting distance between these two modules was 150 m. The driver of the SUV approached at 30 km/h, while the participant input the bus number. When the bus module buzzed and the warning LED flashed, the distance between the bus module and the user module was recorded. This test was repeated five times for each participant.

Test 3 took place on a city road in sunny weather. Before the tests, it took the two participants approximately three to five minutes to learn how to use the user module. The participants held the user module at a bus stop. The experimenter was on board a bus equipped with the bus module, a few stops before the participants. The driver of the city bus was driving normally, while the participant was inputting the bus number. When the bus module buzzed and the warning LED flashed, the distance between the bus module and the user module was recorded by the experimenter. These tests were repeated five times for each participant.

We defined the success of interactive wireless communication as the user module buzzed in response to the bus module.

## 3. Results

The results of the tests are shown in Table 1. In all three tests, the success rates of the interactive wireless communication, recognition of the bus’ arrival, and boarding of the correct bus were all 100%. The maximum wireless communication distances of these tests were below 100 meters. In Test 1, the average, maximum, and minimum wireless communication distance was the longest when the weather was sunny, followed by cloudy days and then by rainy days. The minimum communication distance was greater than 12.1 meters in sunny and cloudy weather, but failed in rainy weather. In Test 2, the minimum wireless communication distance was longer than 12.1 meters for both participants. In Test 3, the minimum wireless communication distance for both participants was shorter than 12.1 meters, but the average distance was longer than 12.1 meters for participant B. The bus traveled at a speed of approximately 20 to 30 km/h.

**Table 1:** Results of Test 1 to Test 3.

Test	One			Two		Three	
Participant	Experimenter	Experimenter	Experimenter	Visual Impaired A	Visual Impaired B	Visual Impaired A	Visual Impaired B
Weather	Sunny	Cloudy	Rainy	Sunny	Sunny	Sunny	Sunny
Vehicle	Motorcycle	Motorcycle	Motorcycle	SUV	SUV	Bus	Bus
Speed (km/h)	30	30	30	30	30	20–30	20–30
**Success rate (%)**							
Wireless communication	100	100	100	100	100	100	100
Recognizing arrival of the bus	-	-	-	100	100	100	100
Boarding the correct bus	-	-	-	100	100	100	100
**Wireless communication distance (m)**							
Minimum	35.0	15.0	8.0	15.0	25.0	5.0	10.0
Maximum	45.0	35.0	15.0	50.0	33.0	15.0	25 .0
Average (mean±SD)	40.0 ± 5.0	26.7 ± 7.4	11.0 ± 2.7	29.4 ± 13.5	29.6 ± 3.2	10.0 ± 5.0	16.7 ± 7.6

Notes: SUV, sports utility vehicle. SD, standard deviation.

## 4. Discussion

Our low-cost system facilitated successful communication and feedback between visually impaired people and bus drivers. Using the interactive communication modules, visually impaired people can give information to bus drivers actively and board the correct bus easily. Bus drivers can receive notification early and send feedback to passengers automatically. This system created direct and fast wireless communication between visually impaired people and city buses drivers.

The interactive wireless communication was successful in all tests, meaning the concept of FDMA was suitable for this system. However, in Test 1, wireless communication was substantially influenced by atmospheric attenuation. We also observed that when the antenna of the user module is perpendicular to the ground, the wireless communication distance was the longest. The position of the user module changed the direction of the antenna and influenced the wireless communication distance. This might be the reason for differences in the minimum and maximum wireless communication between participants in Tests 2 and 3. Further improvement of the wireless communication is necessary before commercialization of this system is possible.

In Test 3, the minimum wireless communication distance for both participants was less than 12.1 meters. Compared with the results of Test 2, the wireless communication distance in Test 3 was an average of 54.8% shorter. This result might be attributed to the bus windows being closed and to the city roads being surrounded by large buildings. Although the wireless communication distance was insufficient, both participants were able to successfully board the correct bus. We observed the bus travelling at a speed of approximately 20 km/h when approaching the stop. The stopping distance of a bus at 20 km/h on dry roads is approximately 6.0 meters [26]. The driver was able to stop the bus quickly and easily.

In Taipei, bus drivers are inclined to stop the bus at bus terminals only once passengers have indicated that they want to board. Therefore, visually impaired people need to hold a large piece of paper with the bus number written on it at bus terminal, or ask other passengers to inform them the arrival of the bus. We interviewed the bus drivers about the experience of using the bus module after Test 3. The bus drivers believed this interactive communication aid system could alert drivers to visually impaired people who are waiting for the arrival of a bus and reduce the drivers’ mental workloads.

After Test 3, the participants provided their suggestions for the interactive wireless communication system. Both of the visually impaired participants believed the system would help to differentiate between several buses arriving simultaneously. They agreed that the bus or bus-stop voice reporting systems are not suitable for a noisy environment in Taipei. If the visually impaired can give information to bus drivers actively, they would be less likely to be ignored at bus stop. The participants also wished the market price of the user module is less than 100 U.S dollars. Regarding the interface of the user module, they agreed that it was an easily operated system. Regarding the hardware design, they suggested a smaller user module with voice keypad and voice feedback. They also mentioned about the combination of the user module and the smart bus stops. If succeeded, they don’t have to bring the user module all the time.

Further improvements that need to be made to the system include: (1) improving the stability of the wireless communication to reduce factors, such as weather and the neighboring environment, affecting the interactive wireless communication distance; (2) upgrading the system to a multi-pair and multi-direction wireless communication system that meets the requirements of a larger group of visually impaired people taking multiple buses at a given time; (3) upgrading the sound feedback of the keypad on the user module to an automated voice keypad and providing voice instructions. Improving these functions will allow for a more fully developed interactive wireless communication system.

## 5. Conclusions

An interactive wireless communication aid system for the visually impaired to use city buses was developed in this study. Results of tests indicated that this system could help users to successfully board their desired buses, using the interactive communication modules, which worked most favorably in sunny weather. The interface design of this system is simple to use and easy to understand. The research demonstrated the feasibility of the proposed system, and provided a reference basis for developing a new system for aiding visually impaired bus users in the future.
